# Caregiver, secondary victim: outcome of caring for patients with COVID -19: a qualitative content analysis study

**DOI:** 10.1186/s12913-023-09319-5

**Published:** 2023-03-30

**Authors:** Safieh Faghani, Fazlollah Ahmadi, Eesa Mohammadi

**Affiliations:** Nursing Department, Faculty of Medical Sciences, Tarbiat Modares University, Tehran, Iran

**Keywords:** Family caregiver, Informal caregiver, COVID-19, Qualitative research, Patient care

## Abstract

**Background:**

Considering the importance of caring for patients with COVID -19 at home and the majority of care being the responsibility of family caregivers, it is necessary to identify and assess the problems during the implementation of patient care in family caregivers. Therefore, the present study was conducted to discover the different consequences of caring for patients with COVID-19 in family caregivers.

**Method:**

Using Purposive sampling, 15 female family caregivers were included in the study. This study was conducted between 2021 and 2022 in Iran. Unstructured face-to-face and virtual interviews were used to collect data until data saturation was reached. Data were analyzed through Granheim and Lundman conventional content analysis approach.

**Results:**

The analysis of data related to the outcome of caring for patients with COVID -19 in family caregivers, led to the extraction of six subcategories: " caregivers experiencing physical symptoms ", "perception of extra pressure and psychological symptoms in the caregiver", "disruption in marital relations", "feeling of homelessness and rejection" and " role pressure due to lack of family support". The subcategories led to the development of the main category "caregiver, the secondary victim", which is experienced by family caregivers during the provision of care for patients with COVID -19.

**Conclusion:**

Family caregivers experience high levels of negative consequences from providing care to patients with COVID-19. Therefore, more attention should be paid to all dimensions of caregiver health such as physical, mental, and marital to provide quality care to patients finally.

## Introduction

The novel coronavirus pandemic (COVID-19) has physically, mentally, economically, and socially damaged the lives of many people around the world. This has taken place mainly due to its rapid spread, high mortality rate, complete change in social habits, and incalculable economic impacts, and consequently, it has put a lot of pressure on health systems [1]. Moreover, a large number of patients and referrals to hospitals have increased the number of hospitalizations, treatment costs, and work pressure on nurses [2, 3]. Therefore, to reduce the pressure on health systems and care for patients with mild to moderate symptoms in the current healthcare situation, every home is a potential hospital [4]. At the same time, informal providers of home services are essential human resources that improve community health care capacity [5, 6].

According to the statistics of the World Health Organization, Iran is one of the countries with the highest number of cases of this disease, with 7,560,629 infected and 144,672 deaths by December 2022 [7]. In Iran, during some waves of COVID-19, the number of patients admitted to the hospital with COVID -19 was such that the hospitals were unable to accept new patients due to the lack of workforce, physical space, and protective and treatment equipment, and many patients with mild to moderate symptoms were referred home to receive care by family caregivers [8]. Also, some families tried to take care of their patients at home due to concerns about hospital costs or the patient's health.

Home care providers are usually referred to as formal or informal caregivers. Most informal caregivers are family members such as spouses and adult children of the care recipient. Therefore, informal caregivers are often called family caregivers [9]. Since the announcement of the COVID-19 pandemic, much research has been published on the experience and psychological impact of COVID-19 on frontline health care workers [10–12]. However, the favorable or unfavorable effects of caring for a patient with COVID-19 on informal caregivers have been largely ignored so far. COVID-19 disease and its complications reduce people's mental health. This disease can disrupt the patient's social and family relationships, and not only does it disrupt the patients' lives but it also affects the caregivers [13]. During the COVID-19 pandemic, the quality of life of family caregivers was greatly affected. Family caregivers provide actions related to symptom management, treatment, and physical, psychological, and social care of the patient [14]. However, they encounter many problems due to the limited education and resources available as well as their lack of knowledge about this emergency disease, the natural course of the disease and how to care for patients. They are also likely to experience more challenges such as uncertainty, separation from others, and fear of the disease and its consequences, they face compared to caregivers of people with other diseases [15, 16]. In addition, many family caregivers in Iran face limited access to medical facilities like shortages and high cost of some drugs needed for COVID-19 patients and lack of oxygen devices, and dissatisfaction with the behavior of medical staff such as Insufficient explanation about the patient’s condition [17]. Family caregivers perform most of the medical and nursing tasks and experience negative effects. Almost half of the caregivers who perform medical or nursing duties worry about making a mistake [4]. Studies have shown that family caregivers show disturbing behaviors and health habits [18] and experience high levels of psychiatric disorders such as stress, anxiety, depression [19, 20], and care burden [21]. Although informal caregivers are a core component of health and social care systems, their experiences, health impacts, and caregiving needs have been largely overlooked during COVID-19 [22].

Considering that, on the one hand, the need for family caregivers increased even more during the COVID -19 and caregivers have an influential role in helping patients with mild to moderate symptoms and reducing pressure on the healthcare system, on the other hand, the review of studies shows that limited qualitative studies have been conducted on the outcomes of caregiving in family caregivers of patients with COVID-19, such as exploring the psychological experience of hospitalized family caregivers with COVID-19 [23] and most studies focus on the effects of the pandemic on family caregivers of adults living in the community who need help because of frailty or illness. Considering that the special features of COVID -19 disease due to severity, lethality, mode and speed of transmission [24], the care of these patients by formal and informal caregivers with many and different challenges such as separation from others, guilt, fear of the disease and its consequences are associated with uncertainty and stress after the accident [16, 25] and it is possible that the experiences of caregivers of patients with COVID-19 in the care process and its consequences are different from those of caregivers of other diseases [26–28].

Therefore, addressing the problems of family caregivers of patients with COVID -19 is necessary to provide appropriate treatment and social support and ensure continuity of care, which requires understanding the consequences of their care [29]. Also, quantitative research does not have the necessary flexibility and depth to explore the experience of a phenomenon. Therefore, since this study aimed to discover the consequences of the experience of caring for patients with Covid-19 in family caregivers, the conventional qualitative content analysis method was used.

## Method

The present study was qualitative research using content analysis with a conventional approach. Conventional content analysis can detect people's perceptions concerning Consequences of care [30].

### Participants and research environment

This qualitative study was conducted from November 2021 to April 2022. Participants were 15 female family caregiver providers purposively selected from the health center in Iran, and they were primarily responsible for the care of COVID -19 patients at home. First, a list of phone numbers of patients infected with COVID-19 was prepared from the community health center in Sari in Mazandaran province of Iran, which receives all the statistics of corona patients from all over the province., and then the contact information of home caregivers was obtained, Iran. Despite considering the principles of purposeful sampling, we tried to select participants by using maximum variation sampling in terms of age, educational levels, occupational status, and residential location. The inclusion criteria were having experience in caring for a patient with COVID-19, willingness to conduct interviews, being caregivers 18 years and older, and being able to communicate. In addition, all patients had a definite diagnosis of COVID -19 with justified medical tests. Exclusion criteria included not completing the interview, refusing to record the interview, and being infected with COVID -19 on the day of the interview.

### Data collection

Data were collected using unstructured in-depth interviews. The time, place, and manner of face-to-face or telephone interviews were determined by family caregivers. All face-to-face interviews were conducted at the residence of family caregivers and only in the presence of the caregiver and the first author. Initially, two pilot interviews were conducted to develop an interview guide. Three of the authors assessed the integrity and the accuracy of these two interviews and the interview guide was developed. The interviews were conducted by asking open-ended questions to allow participants to share their experiences freely. An attempt was made to first gain the interviewee’s trust by introducing themselves, the research objectives, and the method of conducting the interview. After obtaining informed consent, the demographic characteristics of the interviewee such as age, educational status, marital status, and care experience were recorded. Then, the interview started with open-ended and general questions, such as: "How did you take care of your patient?", " What problems did you face while taking care of your patient?" Based on the answers, more specific questions were presented in line with the main question of the research to discover the consequences of care for caregivers (Table 1). In addition, probe questions or clarifiers like "You mean?", "Can you explain more?" What do you mean? Can you give a practical example of this experience? "It was asked.

**Table 1:** Interview Question Guide

No	Questions
1	How did you take care of your patient?
2	What problems did you face while taking care of your patient?
3	Did your physical health change during patient care? Explain
4	Was your mental and emotional health different during patient care compared to before? Explain
5	Explain more about the concern you mentioned
6	Did other family members help you in caring for the patient? Explain
7	Were you supported by the family to take care of the patient? Explain
8	What impact did patient care have on your personal and family life? Explain
9	Did your social relationships change as a result of caring for your patient during care? Explain
10	How did your relatives and acquaintances treat you during the care of your patient? Explain
11	What problems and fears did you have during patient care? Explain

The duration of the interviews was from 25 to 80 min. Each interview was recorded using an MP3 player after obtaining permission and consent from the participants. Each interview was immediately transcribed verbatim. Data collection was continued up to data saturation, i.e., lack of access to new codes and concepts in the next interview and data analysis revealed repeated themes, with no new findings or changes [31] which was achieved after 13 interviews. Nonetheless, two more interviews were held to ensure data saturation, and hence, data collection was finished after 15 interviews**.** One of the family caregivers refused to participate in the study due to the long participation time (40–45 min). Due to compliance with quarantine conditions and public health protocols, half of the interviews were conducted virtually (phone calls) by the first author.

### Data analysis

In this research, the content analysis with a conventional (inductive) approach of Granheim and Lundman which includes three stages: selecting meaning units, condensing and coding, and creating categories and themes on various levels was used for data analysis. First, the information is converted into text format, and after each interview, the researcher will transcribe the information word by word and convert it into text format. Then, the unit of analysis refers to the main unit of the written text, which should be classified during content analysis. The unit of analysis in this method is the entire text of each interview. For this purpose, before starting the analysis, the entire text was read several times. Later, coding units were selected as the main part of the content analysis. The entire text was considered as a unit of analysis and classification was done, and in the next step, the codes were classified and the ability to discriminate between classes was done using the continuous comparison method. In the next step, the coding test and the stability of the coding were done and finally led to the creation of classes and abstract themes [32]. “Credibility”, “conformability”, “dependability” and “transferability” were assessed to confirm the accuracy of the study results [33]. To ensure credibility, prolonged engagement with the data and member checking was used. After the extraction of the initial codes, the coded texts were provided to some family caregivers for checking the accuracy of the interpretations. Confirmability was ensured through peer checking, in which one external peer experienced in qualitative research assessed some of the interviews and the corresponding codes and categories and confirmed the accuracy of the data analysis we used the consolidated criteria for reporting qualitative studies (COREQ) to write this article. Dependability was also ensured through data analysis by the study authors who shared their results and reached an agreement on data analysis in several meetings to confirm the consistency of the categories with the family caregiver's statements. Sampling with maximum variation, descriptions of participants’ characteristics, and comparison of the study findings with the findings of other studies were also performed to ensure transferability.

## Result

Family caregivers included 15 women with an age range of 24 to 58 years. In terms of education level, 3 caregivers had a high school education, 2 caregivers had a diploma, 2 caregivers had an associate degree, 5 caregivers had a bachelor's degree, and 3 caregivers had a master's degree. Both single and married caregivers were included in the study. In terms of occupation, they were both housewives (8 persons) and employed (7 persons). Caregivers were mostly wives (9 cases), children (4 cases), and mothers (2 cases) of the patients. After analyzing the data of 15 interviews, 120 initial codes were extracted, which reached 95 main codes after removing duplicate codes and re-reviewing and reducing codes and merging similar codes.

Finally, the data analysis led to the extraction of six sub-categories: " caregivers experiencing physical symptoms", "perception of extra pressure and psychological symptoms in the caregiver", "disruption in marital relations", "feeling of homelessness and rejection" and " role pressure due to lack of family support". The subcategories led to the development of the main category "Caregiver, the secondary victim" (Table 2), which is experienced by family caregivers during the provision of care to patients with COVID -19. This concept expresses the effects and sometimes complications of patient care in the caregivers of patients with COVID -19. In the care process, many caregivers are involved or suffer from physical, mental, sexual, emotional, and financial problems and sufferings due to their comprehensive attention to the patient and sometimes neglecting themselves, which makes the process of care exhausting for them. And its effects are present in caregivers even long after their patient has recovered.

### Caregivers experiencing physical symptoms

**Table 2:** The products of data analysis in this study

Code	Subcategory	Category
Excessive physical fatigue caused by housework, disinfection and patient care (p2,4,10,11,12,13,14) The feeling of damage to the lungs due to the excessive use of disinfectants (p3,5,13,14) Sleep disturbance due to stress and worry about disease symptoms and loss of the patient (p1,5,9,11,13,14)	caregivers experiencing physical symptoms	Caregiver, secondary victim
Fear, stress, and increase in obsessive behaviors due to the possibility of the family being infected with Corona again (p9,10,12,14) The feeling of impasse and helplessness in care due to the continuation or progression of symptoms despite care (p7,9,10,11)	perception of extra pressure and psychological symptoms in the caregiver	
Discomfort from the disorder in sexual relations with the spouse (patient) (p10,11,12,) The need to establish an emotional and physical relationship with the spouse (patient) (p11,15,9)	disruption in marital relations	
The feeling of being rejected by others due to the fear of being infected and contagious like cholera and plague (p2,9,13) Not helping others because of the fear of the disease being contagious (p1,5,6,7,8,9,11)	the feeling of hopelessness and rejection	
Complaining and getting nervous about the patient's poor cooperation in receiving care (p2,3,4,10,11,13,14) Anger from the high expectations of the family during the caring of patient care (p3,8,13)	Role pressure due to lack of family support	

In the conditions of quarantine and caring for patients with COVID-19, in addition to caring for the patient, caregivers have other responsibilities such as taking care of the needs of other family members, accompanying children in online classes, and shopping outside the home, which has led to an increase in their workload. With the increasing burden and stress on caregivers, many of them do not have enough ability and opportunity to use the self-care methods and coping strategies that they had before the COVID -19 pandemic. A family caregiver said in this regard:

" When you are alone and you have to take care of alone, your duties increase, housework, taking care of the sick, online classes for children, and shopping. I had completely forgotten myself, sometimes I would not eat anything for the whole day. I was so involved in care that I forgot to pay attention to myself, during that time I forgot to take levothyroxine pills and the pain in my leg increased and I realized that my hypothyroidism has relapsed again (Participant 8).

Regarding the complication caused by the pressure of patient care, a caregiver said:"I had severe back pain, very bad hand pains, and a lot of fatigue. This fatigue was different from all the fatigue I experienced… I call it corona fatigue" (Participant 13).

Also, many caregivers experience sleep disorders (quantity and quality) due to stress and worry about the symptoms of the disease, loss of the patient, and continuous monitoring of the patient at night. A family caregiver said in this regard:

"I didn't sleep at all during that time, it's not that I didn't sleep, but I couldn't sleep, I was sleeping all the time, I had nightmares that my mother was dying. " (Participant 10)

### Perception of extra pressure and psychological symptoms in the caregiver

Caregivers often put the patient's needs before their own. They sacrifice their time, energy, and physical and emotional needs, which leads to the fact that caregivers, in addition to physical injuries, suffer many psychological injuries such as depression, anxiety, and stress. Additionally, they deal with high levels of negative emotions including as helplessness, hopelessness, experience fear and worry, irritability, feeling isolated or abandoned by others, etc. A participant said:

"I used all my power to take care of him, even more than I could.... I tried to take good care of his despite all these pressures. This mental pressure bothered me more. The pressure I endured, honestly, I've never experienced anything like this before. " (Participant 6)

In addition, some caregivers experience depression following the caregiving process and the quarantine period. The family caregiver talked about this:

"Psychologically, it really puts pressure on a person, not only in that situation. After Corona, I felt that both myself and my wife were very withdrawn. I was no longer cheerful and happy. " (Participant 11)

### Disruption in marital relations

In the process of illness and caring for a patient with COVID -19, caregivers experience challenges such as refusal or fear of establishing an emotional and sexual relationship with their spouse in their marital relationship, and the caregivers express their distress over this issue. A caregiver said about this:

" Normally, I didn't have much contact with my husband during the day, but during that period, I felt like I wanted to hug him more and sit next to him... I needed him emotionally. But due to the quarantine and his illness, I couldn't be with him very much and it was hard for me, it was sad " (Participant 11)

Another caregiver said about neglecting his sexual needs due to the occurrence of sexual problems in his wife and fatigue and stress caused by caregiving in herself:

"My husband had physical problems in sexual relations, for which he was upset, so that his self-confidence does not decrease, I did not request him and I did not tell my needs to my husband. Caring fatigue and financial pressures also had a negative effect on my marital relationship (Participant 12)."

It has been seen that after contracting and recovering from the disease, the person becomes very weak and cannot have a relationship. Sometimes both the patient and the caregiver do not feel good psychologically and emotionally and do not want to establish a marital relationship. A family caregiver related to this issue said:

"During those two weeks and even two months later, my spouse and I were so mentally tired that we had no desire to have a marital relationship, and both my spouse and I were upset" (Participant 10).

Also, another caregiver said:

" As if my husband's illness affected me and I had no desire at all. Since then and even now, I don't enjoy my sex." (Participant 15)

### The feeling of homelessness and rejection

Another consequence of the process of caring for a patient with the coronavirus, due to the contagiousness of the disease and the quarantine conditions for caregivers, is the feeling of loneliness and rejection by others in the process of care. On the other hand, according to the caregivers, some relatives, neighbors, and even some family members show inappropriate and unsupportive behavior and run away from them. They try to avoid accepting them in public and confronting them even after the patient's recovery. The caregiver said:

"About a month had passed since my husband's illness, it was my niece's birthday, everyone except us... telling us not to come... I mean, we wanted to forget the bitter and difficult days. They didn't allow us to deal with them. It was worse than the days of illness... " (Participant 11)

Also, some caregivers feel rejection behaviors even by the treatment team, especially nurses, due to the refusal to provide services at home due to the patient's infection with COVID-19. A family caregiver explained this:

"We asked the home care nurse for help to inject medicine at home, but she didn't come either and frankly speaking, we don't do it for a patient with corona..." (Participant 7)

### Role pressure due to lack of family support

In the care process, caregivers are trying hard to balance the needs of healthy and sick family members, and due to the nature of caring for a patient with COVID -19 and the lack of help from others, they experience an increase in the burden of responsibility at home and may not be able to maintain this balance, because the caregiver must not only respond to the needs of the patient but also respond to many family problems and needs. A family caregiver said:

"My personal and emotional life with my husband was in trouble. I was angry with my sister and brother. They expect me to do all the care, housework and shopping on my own. Patient care was becoming difficult and out of control for me. " (Participant 13)

Also, some caregivers, contrary to the expectation of support from their families, feel angry because of the disconnection of the family with the caregiver and the patient. A participant said:

"I was angry with the family, they left us in general and cut ties with us for two months. They did not cooperate with me and did not understand my problems in caring for the patient. (Participant 15)

## Discussion

This study was conducted to understand the consequences of caring for a patient with COVID -19 in family caregivers. The results showed that the caregivers of patients with COVID-19 are the secondary victims of this disease. This means that due to the characteristics of this disease and the need for quick, continuous, accurate care related to the patient's symptoms, caregivers experiencing physical symptoms by sacrificing, neglecting, and sacrificing their own needs, even physiological needs to care for the patient.

One of the results of the present study was the caregiver's involvement with her own physical symptoms and complications. Many caregivers suffer from disturbed sleep quality and quantity due to continuous monitoring of the patient even during night sleep. The results of a systematic review study showed that 76% of caregivers reported poor sleep quality such as short sleep duration and frequent awakenings at night, and this ratio is significantly higher for female caregivers compared to male caregivers [34]. Therefore, in the context of a pandemic, interventions for carers' physical problems and improved sleep quality should be provided when necessary. This may include providing caregiver support to reduce caregiver burden, increasing social support, and providing health education materials that provide strategies for identifying and seeking social support [35]. Caregivers in the present study also experienced excessive, progressive, and chronic fatigue due to caregiving pressure and the resulting stress. According to the results of the present study, a cross-sectional study was conducted in Iran to investigate the amount of care burden on family caregivers of COVID -19 patients discharged from the hospital. The results showed that the intensity of care burden experienced by family caregivers was moderate and the most intensive burden of care that they experienced was a physical burden [36]. Furthermore, one of the results of the study by Malmir et al. (2022) which examined the challenges of elderly family caregivers with multiple chronic diseases showed that caregivers are forced to move the elderly for various tasks, which can cause physical problems such as back pain, wrist pain, pelvic pressure, body pain, and bruises for caregivers [37]. Also, in a study conducted on the caregivers of different patients during the COVID-19 pandemic, the results showed that family caregivers are affected by a variety of challenges and additional burdens. Therefore, support and relief services should be tailored to the needs of family caregivers, including promoting mental health, providing protective materials, and taking into account information needs, and should be user-centered and accessible as much as possible, avoiding unnecessary administrative procedures [21]. Therefore, the physical health status of family caregivers, especially caregivers of patients with COVID -19 and other contagious pandemics that we may witness, which is especially in many cases due to unprincipled management of the care process, requires the serious attention of care providers So that they do not become secondary victims of COVID -19.

These are only a part of the problems and sufferings that the caregivers suffer during the provision of patient care. They understand and experience double pressure and various psychological symptoms such as anxiety, depression, loneliness, anger, and aggression for various reasons including care during the quarantine period, fear of the possibility of re-infection of the patient and family, and fatigue and pressure caused by care. Related to the results of the present study, the results of other studies also showed that the home caregivers of patients experience higher levels of stress, anxiety, and depression symptoms during COVID-19 [38, 39]. Also, Beach et al. examined the consequences of physical, psychosocial, and financial well-being during the COVID-19 pandemic in family caregivers. The results showed anxiety, depression, fatigue, sleep disorder, less social participation, lower financial well-being, increased food insecurity, and increased financial worries. And in general, the care challenges were intensified by the COVID -19 pandemic [40]. In the study of Irandoost et al., who explained the problems faced by Iranian housewives during the quarantine period of COVID -19, the results of the study showed that they experience personal, family, and social problems such as living with fear and anxiety, low mental health, violence, and conflict in the family and tension in managing family members [41]. As a result, it is necessary to implement providing psychological services as one of the effective methods to reduce the burden of care for family caregivers of COVID -19 patients [42].

Also, the results of the present study showed that caregivers in marital relationships with their spouses, on the one hand, due to the effects of fatigue, fear of the patient's death, financial pressures on the caregiver's sexual performance during patient care, and on the other hand, due to the occurrence of sexual problems in the patient, have emotional problems and disruption in marital relations. In this regard, the results of the study of Luetke et al. (2020) showed that 34% of couples were not satisfied with their sexual relations during the 2019 coronavirus pandemic. Also, people who were more afraid of COVID -19 felt more emotionally lonely and they spent little time with their partners [43]. Also, Feng et al. (2021) investigated the changes in sexual relations during the outbreak of COVID -19 in research. The findings indicated a 42% decrease in sexual activity during quarantine [44]. On the other hand, contrary to the results of the present study, the study by Williamson (2020) showed that people's marital satisfaction levels had not changed much. Only in families where the spouses did not fulfill their duties and destroyed each other's privacy, marital conflicts were observed [45]. This difference in the results can be because the above studies were conducted in the normal population, but family caregivers experience different and difficult challenges that can have a double negative effect on their marital relationships.

Also, caregivers experience lonely care while taking care of their patients due to special disease characteristics such as the rapid transmission of the disease, its contagiousness, and high lethality. And they are always upset and complain about being rejected by others and in many cases even the family. The results of Faghani et al.'s study (2023) showed that the family caregivers of patients with COVID-19 provided selfless, continuous, and comprehensive care, accompanied by fear and loneliness, which increased the quality of care and life for patients, while the care was accompanied by fear and loneliness can have harmful effects on both family caregivers and the quality of care in the long term [46]. And one of the remarkable findings, different and contrary to the Iranian culture in the present study, was experienced role pressure due to lack of family support. The results of Hindmarch et al.'s study (2021) showed that family caregivers experienced negative consequences including social isolation (66%), stress (63%), and reduced quality of life (57%) during the COVID -19 pandemic [47]. In this regard, the results of Akbarbegloo et al.'s (2022) study, which examined the lived experiences of family caregivers of patients with COVID -19 during a phenomenological study, showed that family caregivers experience isolation, difficult conditions of care, concealment of illness, and insufficient support during care [48]. Also, in the phenomenological study that Asgari et al. conducted to investigate the experiences of family caregivers of an Iranian patient with COVID -19 at home, the results showed that caregivers feel helpless, lonely, and rejected by others due to the nature of the disease and its severity [49]. Also, in line with the present study, the results of Irandoost et al.'s (2022) study, which examined the lived experiences and challenges of the families of the victims of COVID -19, indicate the experience of many challenges in the care of a patient with COVID-19, such as being rejected by others and disruption in family life was in families [17].

Therefore, the possibility of person-to-person transmission, the high level of ignorance about the disease, the fear of ambiguity and lack of knowledge, and the constant changes in relevant regulations and recommendations lead to the stigma of COVID-19 and exclusion. These negative consequences, including the concealment of illness, lead to delays in seeking health services, stress, and related socioeconomic consequences. Therefore, necessary measures should be taken in this regard. In particular, the spread of stigma and rejection should be prevented by measures to increase awareness and ensure access to reliable information [50].

### Study limitation

One of the limitations of the present study was that our participants were only selected from the female caregivers of COVID- 19 patients because the majority of family caregivers in Iran are women. Also, although an effort was made to have maximum diversity in the participants, it was impossible to cover all the diversity in the social and cultural context of Iranian society. Another limitation is that some of the interviews were conducted over the phone, which may have caused the participants not to pay enough attention and focus, and the researcher did not have access to their non-verbal reactions.

## Conclusion

The findings showed that providing care to patients with COVID -19 has an impact on various aspects of family caregivers' lives. Therefore, great attention should be paid to the caregiver's physical, mental, marital, and family health to provide high-quality care to the patient. Because these pressures and complications caused by care may reduce the amount and quality of patient care, and in the long term, caregivers may need intervention, counseling, and follow-up, and as a result, an additional burden will be imposed on the health system.

## Data Availability

The datasets generated during and analyzed during the current study are not publicly available due to ethical restrictions but are available from the corresponding author on reasonable request.
